# Multimodal single-neuron, intracranial EEG, and fMRI brain responses during movie watching in human patients

**DOI:** 10.1038/s41597-024-03029-1

**Published:** 2024-02-16

**Authors:** Umit Keles, Julien Dubois, Kevin J. M. Le, J. Michael Tyszka, David A. Kahn, Chrystal M. Reed, Jeffrey M. Chung, Adam N. Mamelak, Ralph Adolphs, Ueli Rutishauser

**Affiliations:** 1 Department of Neurosurgery, Cedars-Sinai Medical Center, Los Angeles, CA USA; 2 Division of the Humanities and Social Sciences, California Institute of Technology, Pasadena, CA USA; 3 Division of Biology and Biological Engineering, California Institute of Technology, Pasadena, CA USA; 4 Department of Neurology, Cedars-Sinai Medical Center, Los Angeles, CA USA; 5 Center for Neural Science and Medicine, Department of Biomedical Sciences, Cedars-Sinai Medical Center, Los Angeles, CA USA

**Keywords:** Cognitive neuroscience, Long-term memory

## Abstract

We present a multimodal dataset of intracranial recordings, fMRI, and eye tracking in 20 participants during movie watching. Recordings consist of single neurons, local field potential, and intracranial EEG activity acquired from depth electrodes targeting the amygdala, hippocampus, and medial frontal cortex implanted for monitoring of epileptic seizures. Participants watched an 8-min long excerpt from the video “Bang! You’re Dead” and performed a recognition memory test for movie content. 3 T fMRI activity was recorded prior to surgery in 11 of these participants while performing the same task. This NWB- and BIDS-formatted dataset includes spike times, field potential activity, behavior, eye tracking, electrode locations, demographics, and functional and structural MRI scans. For technical validation, we provide signal quality metrics, assess eye tracking quality, behavior, the tuning of cells and high-frequency broadband power field potentials to familiarity and event boundaries, and show brain-wide inter-subject correlations for fMRI. This dataset will facilitate the investigation of brain activity during movie watching, recognition memory, and the neural basis of the fMRI-BOLD signal.

## Background & Summary

The most common approach to investigate neural representations of visual stimuli, decisions, and memory in humans has traditionally been to present static stimuli one at a time. With this trial-by-trial experimental design, analysis of neural activity focuses on relating time-locked experimental events in a particular trial to the neural responses they evoke^1^. For example, a question that would be answered this way in the context of intracranial recordings is to compare the onsets of stimuli that contain faces with those that do not in order to examine the neural correlates of face perception^2^. A key unanswered question is whether the representations revealed by trial-by-trial designs generalize to those seen during more realistic continuous experience^3,4^. A major step in this direction has been the study of neural responses while participants watch short video clips^5^. This has revealed, for example, the existence of cognitive boundaries, which mark periods of time when the ongoing narrative is interrupted during a continuous experience, thereby marking the start of a new episodic memory^6–8^. Importantly, the stimulus selectivity of neural responses seen during continuous presentation can differ markedly from that seen during static stimulus presentation^9^. Despite its ecological advantages, significant challenges remain in the analysis of continuous stimulus protocols. These include the challenge of quantifying which time-varying features of the stimulus are being attended (e.g., using concurrent eye tracking), comprehensive annotation of movies for the relevant features (especially ones that are semantically defined, such as emotions), and ways to extract dynamic features beyond those available in individual frames (notably, events solely inferred from the context, such as anticipating a person when a door begins to open). Here, we provide a comprehensive multi-modal dataset to foster the further development of methods to examine neural activity during movie watching, and we additionally provide data from trial-by-trial responses (in a separate memory task) to enable direct comparisons between continuous movie-evoked activity with more traditional trial-by-trial designs.

A second major question in neuroscience is the neural basis of the fMRI-BOLD signal in general, as well as whether the neural basis of fMRI-BOLD is different during continuous as compared to static experimental designs. A key contribution to our understanding of the fMRI-BOLD signal has come from concurrent fMRI and single-unit electrophysiology in monkeys^10^, an approach not possible in humans. However, these two modalities can be obtained at separate times, in the same patients and using the same stimuli^11–14^. Here, we provide fMRI data from a subset of the same participants from whom we later also recorded electrophysiology, watching the same movie in both conditions. This dataset is therefore a valuable opportunity to compare fMRI-BOLD and invasive electrophysiological activity in the same participants in the same task.

**Fig. 1:**
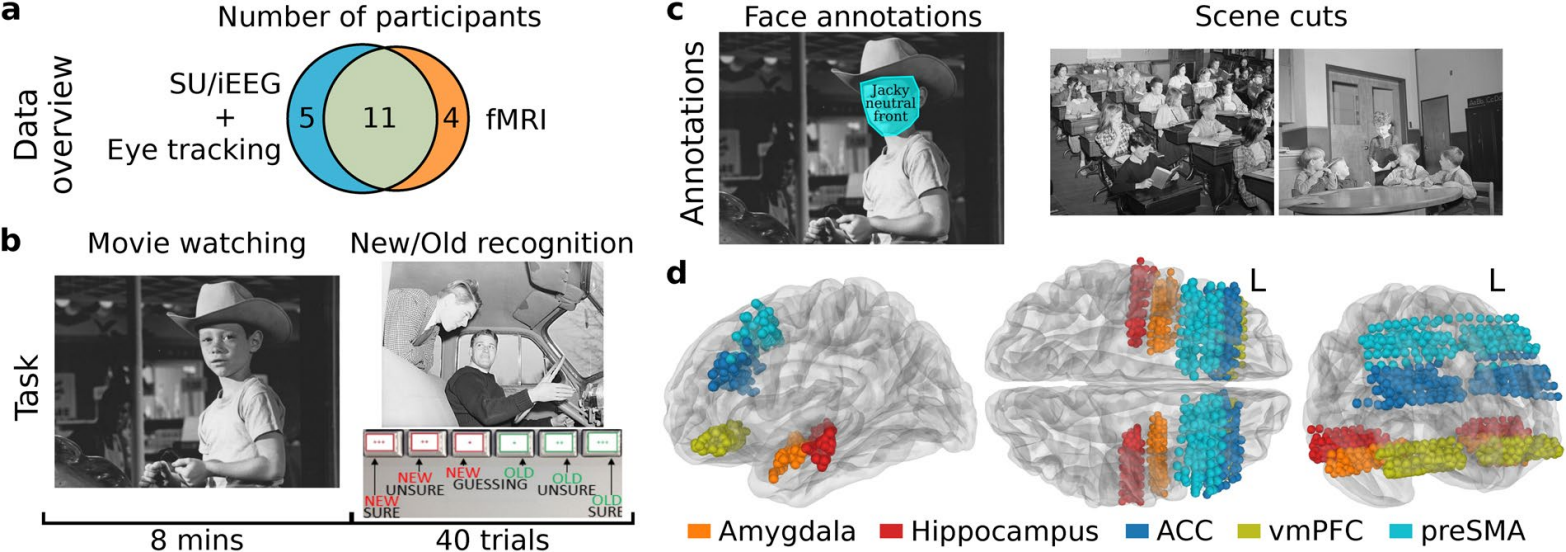
Overview of data and experiment. (**a**) Data overview with the number of participants for each brain recording modality used in the study. (**b**) The task included a movie watching phase first and then a recognition phase (omitted for fMRI). In the movie watching phase, participants watched an audio-visual movie, and in the recognition phase, they viewed 20 novel and 20 familiar movie frames, identifying each image as new (novel) or old (familiar) using a confidence rating scale. (**c**) Manual annotations of movie stimulus. Face areas, emotions, and head pose were provided for each video frame with a face. Scene cuts were annotated to provide information on the start/end time and type of cuts. Due to copyright restrictions of the movie “Bang! You’re Dead”, the visualizations are shown using royalty-free images. (**d**) Recording locations across the patients are shown in the template structural atlas MNI152NLin2009cAsym^39^.

This dataset consists of data from a total of 20 participants (Fig. 1a and Table 1). Of these participants, 11 underwent both fMRI scanning and depth electrode recordings. The stimulus that participants watched is an 8-min long excerpt of Alfred Hitchcock’s “Bang! You’re Dead” movie (Fig. 1b, left). This exact clip has been used repeatedly in neuroimaging work, thereby facilitating comparison to prior work and utilization of the extensive annotations that already exist for this movie^15–17^. While movie viewing was passive, participants subsequently performed a recognition memory task (Fig. 1b, right). This task was intentionally designed as a classic trial-by-trial design to allow direct comparison of neural responses to continuous versus trial-by-trial protocols. During this task, individual frames extracted from the movie were shown while patients performed a recognition confidence judgment (also providing a metric of how well they attended to the movie in the first place). We provide annotations of faces and scene cuts (Fig. 1c). At the electrophysiological level, we provide fully spike-sorted single neurons (1450 neurons in total), local field potential (LFP) activity recorded from the same microwires that were used to record single neurons, and intracranial EEG (iEEG) activity from all clinical macroelectrodes along the shaft of the depth electrodes, providing substantial additional anatomical coverage. All fMRI data were acquired prior to implantation and are whole-brain. The participants included in this study had hybrid depth electrodes targeting the medial temporal lobe (amygdala and hippocampus), and the medial frontal cortex (anterior cingulate cortex, ACC; pre-supplementary motor area, preSMA; and the ventral medial prefrontal cortex, vmPFC). Depth electrodes were implanted in an orthogonal approach, providing coverage of areas such as the dorsolateral PFC (dlPFC), ventrolateral PFC (vlPFC), and medial temporal gyrus (MTG) at the level of iEEG. Participants’ gaze was monitored only during intracranial recording sessions, and we provide the raw gaze position. For the fMRI data, we provide functional data and structural T1 and T2 scans. The data is packaged in two standardized data formats: all data recorded while patients were being monitored with depth electrodes is provided in the Neurodata Without Borders (NWB) format^18^, and all fMRI data is provided in the Brain Imaging Data Structure (BIDS) format^19^.

**Table 1:** Patients.

ID	# of EMU runs	# of fMRI runs	Age	Sex	Epilepsy Diagnosis
P41CS	2	2	21	F	Left Other
P42CS	2	2	25	F	Not Localized
P43CS	2	2	42	F	Left Mesial Temporal
P44CS	1	2	53	F	Right Mesial Temporal
P45CS	NA	2	29	F	Bitemporal
P46CS	NA	2	41	M	NA
P47CS	2	2	32	M	Right Mesial Temporal
P48CS	2	2	32	F	Left Mesial Temporal
P49CS	2	NA	24	F	Left Mesial Temporal
P50CS	NA	2	25	M	Right Temporal Neocortical
P51CS	2	2	17	M	Not Localized
P53CS	2	2	60	M	Bilateral Independent Temporal
P54CS	2	2	59	F	Right Mesial Temporal
P55CS	2	NA	43	F	Right Mesial Temporal
P56CS	2	NA	48	M	Bilateral Independent Temporal
P57CS	2	NA	46	M	Left Other
P58CS	1	NA	32	F	Right Lateral Frontal
P59CS	NA	2	34	M	Left Mesial Temporal
P60CS	1	2	67	M	Left Mesial Temporal
P62CS	2	2	25	F	Right Mesial Temporal
**Total participants: 20**	**Total SU runs: 29**	**Total fMRI runs: 30**	**Mean** (**SD**)**: 37**.**75** (**13**.**86**)	**11 Female**	

Number of EMU and fMRI runs performed, demographics, and pathology.

## Methods

### Participants

We invited 20 patients with intractable epilepsy to participate in two visits: prior to hospital admission (for fMRI), and as in-patients while undergoing invasive epilepsy monitoring with depth electrodes. All electrophysiological recordings took place while patients were in the epilepsy monitoring unit (EMU), and all procedures for electrode implantation, including the anatomical location of the electrodes, were carried out under clinical protocols that were independent of the present study. Eleven participants completed both EMU recordings and fMRI, four participants completed only the fMRI session but then did not proceed with invasive monitoring, and five participants completed only the EMU session but did not enroll in the fMRI part of the protocol (see Fig. 1a and Table 1 for details and demographics). All participants had normal or corrected-to-normal vision. Participation in our research study was voluntary and participants or their legal guardian, if they were under 18 years of age, provided informed consent. All experimental protocols were approved by the Institutional Review Boards of the California Institute of Technology (Caltech; IRB: 16-0692 F) and Cedars-Sinai Medical Center (CSMC; IRB: 13369).

### Task

The task consisted of two experimental sessions: one in the MRI scanner (typically several weeks before the implantation) and one in the EMU following depth electrode implantation (see Table 1 for exceptions regarding participants). In most sessions, participants completed two runs of the experiment (see Table 1 for exceptions regarding sessions) to allow test-retest validation. Each run consisted of two phases: movie watching (both in the EMU and MRI scanner), followed by a recognition memory test in the EMU (Fig. 1b) and an attention test in the scanner. Participants were informed prior to the start of the movie watching phase that it would be followed by a memory or attention test. In the movie watching phase, participants were instructed to watch the audio-visual movie. In the recognition task phase, participants were presented with 20 novel, unseen movie frames (drawn from sections of the original, full version of the Hitchcock movie that were removed for the edited version; see Stimuli section below) and 20 familiar, viewed frames (taken from the edited version of the movie actually presented). Participants identified each frame image as novel or familiar using a confidence rating scale from 1 (novel, not seen during movie watching, sure) through 3 (novel, but most unsure) and 4 (familiar, but most unsure) to 6 (familiar, seen during movie watching, sure); for analysis, ratings of 1, 2, 3 were pooled into the participant’s classification as “novel” and ratings of 4, 5, 6 were pooled as “familiar”. They provided their answers by pressing buttons on an external response box (Fig. 1b). The same set of 80 frame images (consisting of 40 novel and 40 familiar frames) was used in the recognition memory experiment across all participants. The sequence of these images was randomized for each run, ensuring that the set of 40 frames (20 novel and 20 familiar) displayed differed between two runs for each participant, thereby maintaining the novelty of the task.

While the retrieval frames varied between the two runs for each participant, consisting of different subsets, the movie segment shown remained identical. This design was intentional to assess the test-retest reliability of the measured neural signals. However, watching the same movie twice could potentially affect this reliability. Specifically, the familiarity gained from the first viewing could influence participants’ responses in the subsequent run, possibly lowering the reliability. Therefore, this aspect should be considered when assessing the test-retest reliability of neural signals.

The fMRI part of the experiment only contained the movie watching phase (no recognition memory test). Instead, at the end of each movie watching run, participants responded to seven multiple-choice questions about events that took place in the movie, by selecting one of four answer options that assessed their attention and memory for the movie. These questions were drawn from a set of 14 provided by Naci *et al*.^15^. For the first run, we used the seven odd-numbered questions from the original set. The second run utilized the remaining seven even-numbered questions. Participants answered on average 5.97 ± 0.98 (mean ± s.d., across participants and runs) questions correctly. The answers^20^ given by each participant are available on Figshare. The movie presentation and question task were implemented in Matlab using Psychophysics Toolbox^21^. The movie was presented during MRI scanning using a back-projection system viewed through a head coil-mounted mirror. The video projection was 29 cm × 22 cm at a viewing distance of 100 cm resulting in an observed angular size of 16.5° × 12.6°.

### Movie stimulus

The movie stimulus was an 8-min edited excerpt from the television episode “Bang! You’re Dead,” a black-and-white drama directed by Alfred Hitchcock and originally aired in the series “Alfred Hitchcock Presents” (1961). The movie was edited from its original duration of 30 min to 8 min while retaining the essential plot^15–17^. The edit we used was identical to that used in several prior studies, which had demonstrated that this stimulus evokes reliable and reproducible cortical activity across participants^22^.

### Electrodes and electrophysiology

All intracranial recording data in this dataset was acquired from hybrid Behnke-Fried depth electrodes^23,24^ (AdTech Inc.). All recordings were performed with an FDA-approved electrophysiology system (ATLAS system, Neuralynx Inc.). The signal from the microwires was recorded at a sampling rate of 32,000 Hz in broadband (0.1 to 9,000 Hz) and the signal from the macroelectrodes was sampled at 2,000 Hz. Microwire recordings were locally referenced within each recording site by using either one of the eight available micro channels or a dedicated reference channel with lower impedance provided in the bundle.

### Spike detection and sorting

**Fig. 2:**
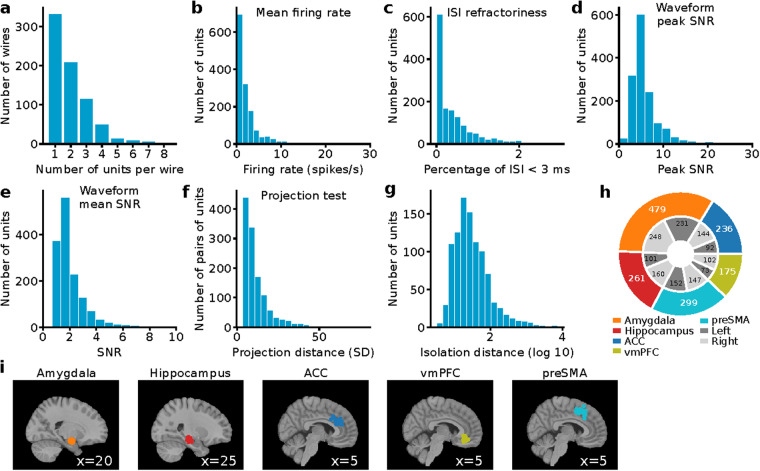
Assessment of recording and spike sorting quality. (**a**) Histogram of the number of units identified on each active wire (only wires with at least one unit identified are counted). (**b**) Histogram of mean firing rates. (**c**) Histogram of proportion of inter-spike intervals (ISIs) which are shorter than 3 ms. (**d**) Histogram of the signal-to-noise ratio (SNR) of the mean waveform peak of each unit. (**e**) Histogram of the SNR of the entire waveform of all units. (**f**) Pairwise distance between all possible pairs of units on all wires where more than 1 cluster was isolated. Distances are expressed in units of standard deviation (SD) after normalizing the data such that the distribution of waveforms around their mean is equal to 1. (**g**) Isolation distance of all units for which this metric was defined. (**h**) Number of cells recorded in each brain area across all the patients. (**i**) Recording locations quantified in (h) visualized anatomically. Each dot is a different electrode in which at least one usable unit was recorded. Shown are sagittal views of the template structural atlas MNI152NLin2009cAsym^39^.

Spike detection and sorting were conducted using the semiautomated template-matching algorithm OSort (version: 4.1)^25^, followed by manual post-processing. Spikes were detected after bandpass filtering the raw signal in the 300–3,000 Hz band. Figure 2 shows spike sorting quality metrics and statistics. For patients that performed multiple runs of the same experiment within the same session, all neurons were sorted together.

### Electrode localization

Electrodes were localized based on a pre-operative MRI and post-operative MRI and/or CT scans as described previously^26^. All electrode localizations were performed in the participant’s native space. In addition, we provide electrode locations in MNI152 coordinates, which we also used for visualization on a structural template atlas (Figs. 1d, 2i). Note that coordinates that appear in white matter or the wrong target structure in Figs. 1d, 2i are due to misregistration to the template brain (all electrode locations shown are confirmed in gray matter in the native space of the subject).

### Eye tracking in EMU

The EyeLink 1000 (SR Research Inc.) eye tracker was used to record monocular gaze position at a sampling rate of 500 Hz using infrared corneal reflection together with a sticker to track head position as described previously^26–28^. The Eyelink’s built-in algorithms were used to classify fixations, saccades, and blinks. We provide the raw gaze position as well as fixations, saccades and blinks, and pupil size (number of pixels inside the pupil contour) throughout the experiment. Eye tracking data was not collected reliably during the MRI scanning and is not part of this data release.

### Eye tracking analysis

We evaluated the congruence of participants’ gaze patterns using temporally segmented gaze heatmaps^29^. For each participant and time segment, two heatmaps were constructed. First, a participant-specific heatmap was generated by applying a two-dimensional Gaussian filter over each gaze point. This filter had a standard deviation equivalent to 1° of visual angle, translating to roughly 33 pixels on our display, estimated by averaging across individual participants’ visual angles. Second, a normative gaze heatmap was generated by aggregating data from all participants and applying the same Gaussian filtering, while excluding the participant being analyzed, thereby mitigating bias in similarity calculation. This normative heatmap served as a reference for the visual saliency during each time segment. The alignment of an individual’s gaze with this normative heatmap for each segment was quantified by calculating the Pearson correlation between their heatmap and the normative heatmap, converting each heatmap into a vector before computation. These correlation coefficients were normalized using the Fisher z-transformation, averaged across segments, and reconverted to provide a mean gaze similarity score, expressed as Pearson’s r. This procedure was then repeated for each participant and each run. We presented our findings using 1-second time segments, though our testing with 0.5 and 2-second segments yielded comparable results.

### MRI data acquisition

All MRI data was acquired at the Caltech Brain Imaging Center using a 3 T scanner equipped with a 32-channel head-receive array (TIM Trio, Siemens Medical Solutions, Malvern, PA). BOLD contrast functional images were acquired during movie viewing with the following parameters: multiband T2*-weighted EPI sequence, TR 1016 ms, TE 30 ms, flip angle 60°, 2.5 mm isotropic voxels, no in-plane acceleration, multiband acceleration factor 4, bandwidth 2404 Hz/pixel, 500 acquired volumes, total imaging time 8 min 28 s. Two runs were acquired of the movie viewing BOLD acquisition for each participant. An additional single-band reference image was generated by the same T2*w EPI sequence for use as an intermediate reference for image registration. Distortion-correction data for the EPI acquisitions employed a pair of phase-encoding polarity-reversed T2w SE-EPI images (TR 4800 ms, TE 50 ms, flip angle 90°) with identical geometry and EPI echo train timing to the T2*w EPI images. Following an MRI system upgrade (Prisma Fit, Siemens Medical Solutions), the BOLD acquisition TR was reduced to 700 ms and the multiband acceleration factor increased to 6. This change would reduce the raw tSNR of all volumes but increase the total number of volumes acquired during the fixed duration movie. This change impacted the BOLD acquisitions for P59CS, P60CS, and P62CS only.

T1w structural images were acquired with the following parameters: 3D MEMP-RAGE with RMS echo combination, TR 2530 ms, TI 1100 ms, TE 1.6, 3.5, 5.4, 7.2 ms, 1.0 mm isotropic voxels, GRAPPA 2 in-plane acceleration, total imaging time 6 min 3 s. T2w structural images were acquired with the following parameters: 3D T2w SPACE sequence, TR 3390 ms, effective TE 390 ms, flip angle 120°, in-plane GRAPPA acceleration 2, bandwidth 650 Hz/pixel, total imaging time 9 min 58 s. A total of four T1w structural images were acquired for each subject, with the exception of P42CS where only three were acquired. Two T2w structural images were acquired for each subject, except for P59CS where three were acquired.

### MRI data preprocessing

The anatomical and functional preprocessing steps (detailed in the “Anatomical Data Preprocessing” and “Functional Data Preprocessing” sections) were generated by *fMRIprep*^30^ and have been included here with minimal modification from the recommended text for clarity and style (see also: https://www.nipreps.org/intro/transparency/#citation-boilerplates).

Results included in this manuscript come from preprocessing performed using *fMRIPrep* 23.1.3^30^ (RRID:SCR_016216), which is based on *Nipype* 1.8.6^31^ (RRID:SCR_002502).

#### Preprocessing of B_0_ inhomogeneity mappings

A total of 2 fieldmaps were found available within the input BIDS structure for this particular subject. A *B*_*0*_ nonuniformity map (or *fieldmap*) was estimated based on two echo-planar imaging (EPI) references using *topup*^32^.

#### Anatomical data preprocessing

All available T1-weighted (T1w) images for each participant were corrected for intensity non-uniformity (INU) with *N4BiasFieldCorrection*^33^, distributed with *ANTs* 2.3.3^34^ (RRID:SCR_004757). An individual average T1w reference image was computed after registration of all INU-corrected T1w images for a given subject using *mri_robust_template*^35^ (FreeSurfer 7.3.2). The T1w reference was then skull-stripped with a Nipype implementation of the *antsBrainExtraction.sh* workflow (from ANTs), using OASIS30ANTs as target template. Brain tissue segmentation of cerebrospinal fluid (CSF), white-matter (WM) and gray-matter (GM) was performed on the brain-extracted T1w reference using *fast*^36^ (FSL 6.0.5.1:57b01774, RRID:SCR_002823). Brain surfaces were reconstructed using *recon-all*^37^ (FreeSurfer 7.3.2, RRID:SCR_001847), and the brain mask estimated previously was refined with a custom variation of the method to reconcile ANTs-derived and FreeSurfer-derived segmentations of the cortical gray-matter of *Mindboggle*^38^ (RRID:SCR_002438).

The following template was selected for spatial normalization: ICBM/MNI 152 Nonlinear Asymmetrical template version 2009c^39^ (RRID:SCR_008796; TemplateFlow ID: MNI152NLin2009cAsym). Volume-based spatial normalization to the MNI152NLin2009cAsym space was performed through nonlinear registration with *antsRegistration* (ANTs 2.3.3), using brain-extracted versions of both the individual T1w reference and the MNI152 T1w template.

#### Functional data preprocessing

For each of the two BOLD runs acquired per participant (across all tasks and sessions), the following preprocessing was performed. First, a reference volume (BOLD Reference) and its skull-stripped version were generated by aligning and averaging the single-band references (SBRef) from the two BOLD runs. Head-motion parameters with respect to the BOLD reference (transformation matrices, and six corresponding rotation and translation parameters) were estimated before any spatiotemporal filtering using *mcflirt*^40^ (FSL 6.0.5.1:57b01774). BOLD runs were slice-time corrected to 0.306 s (0.5 of slice acquisition range 0s-0.613 s) using *3dTshift* from AFNI^41^ (RRID:SCR_005927). The BOLD reference was then co-registered to the T1w reference using *bbregister* (FreeSurfer) which implements boundary-based registration^42^. Co-registration was configured with six degrees of freedom.

Several confounding time-series were calculated based on the *preprocessed BOLD*: framewise displacement (FD), DVARS and three region-wise global signals. FD was computed using two formulations following Power^43^ (absolute sum of relative motions) and Jenkinson^40^ (relative root mean square displacement between affines). FD and DVARS were calculated for each functional run, both using their implementations in *Nipype* (following the definitions by Power *et al*.^43^). The three global signals were extracted within the CSF, the WM, and the whole-brain masks. Additionally, a set of physiological regressors were extracted to allow for component-based noise correction (*CompCor*^44^). Principal components were estimated after high-pass filtering the *preprocessed BOLD* time-series (using a discrete cosine filter with 128 s cut-off) for the two *CompCor* variants: temporal (tCompCor) and anatomical (aCompCor). tCompCor components were then calculated from the top 2% most variable voxels within the brain mask. For aCompCor, three probabilistic masks (CSF, WM and combined CSF + WM) were generated in anatomical space. The implementation differs from that of Behzadi *et al*.^44^ in that instead of eroding the masks by 2 pixels on BOLD space, a mask of pixels that likely contain a volume fraction of GM was subtracted from the aCompCor masks. This mask was obtained by dilating a GM mask extracted from the FreeSurfer’s *aseg* segmentation, to ensure components are not extracted from voxels containing a minimal fraction of GM. Finally, these masks were resampled into BOLD space and binarized by thresholding at 0.99 (as in the original implementation). Components were also calculated separately within the WM and CSF masks. For each CompCor decomposition, the *k* components with the largest singular values were retained, such that the retained components’ time series were sufficient to explain at least 50% of variance across the nuisance mask (CSF, WM, combined, or temporal). The remaining components, accounting for diminishing proportions of variance, were dropped from consideration.

The head-motion estimates calculated in the correction step were also placed within the corresponding confounds file. The confound time series derived from head motion estimates and global signals were expanded with the inclusion of temporal derivatives and quadratic terms for each^45^. Frames that exceeded a threshold of 0.5 mm FD or 1.5 standardized DVARS were annotated as motion outliers. Additional nuisance timeseries were calculated by means of principal components analysis of the signal found within a thin band (*crown*) of voxels around the edge of the brain, as proposed by Patriat *et al*.^46^.

The BOLD time-series were resampled into an MNI standard space, generating a *preprocessed BOLD run in MNI152NLin2009cAsym space*. The BOLD time-series were also resampled onto FreeSurfer r*fsaverage* surface. All resamplings were performed with a single interpolation step by composing all the pertinent transformations (i.e., head-motion transform matrices, susceptibility distortion correction when available, and co-registrations to anatomical and output spaces). Gridded (volumetric) resamplings were performed using *antsApplyTransforms* (ANTs), configured with Lanczos interpolation to minimize the smoothing effects of other kernels^47^ (for native and MNI space). Non-gridded (surface) resamplings were performed using *mri_vol2surf* (for FreeSurfer).

Many internal operations of *fMRIPrep* use *Nilearn* 0.10.1^48^ (RRID:SCR_001362), mostly within the functional processing workflow. For more details of the pipeline, see the section corresponding to workflows in *fMRIPrep’*s documentation (https://fmriprep.readthedocs.io/en/latest/workflows.html).

#### Functional data denoising

The functional data preprocessed by fMRIprep was then denoised by using Python code provided in the GitHub repository associated with the budapest-fmri-data study^49,50^. In this code, ordinary least-squares regression was used to regress out specific nuisance parameters from the functional time series. These nuisance parameters included six motion parameters along with their derivatives, global signal, framewise displacement^43^, the first six noise components estimated by aCompCor^44^, and polynomial trends up to the second order. The denoised data was then used to calculate the metrics of interest, either in native volumetric space or on fsaverage template. No further spatial smoothing or temporal filtering was applied.

### Temporal signal-to-noise ratio (tSNR) in fMRI data

Voxel-wise tSNR values were computed to assess fMRI data quality. We first computed tSNR values in each participant’s native space without applying template normalization. The tSNR was computed for each voxel and for each run by dividing the mean BOLD signal intensity over time by the standard deviation of the signal intensity. Voxel-wise tSNR values were averaged across the two fMRI runs to obtain a single tSNR for each voxel. In addition, to generate a group tSNR map and examine the variation of tSNR across the cortex, we repeated the analysis after first spatially normalizing participant-specific images to the fsaverage template.

### Inter-subject correlation in fMRI data

Inter-subject correlation (ISC) was computed to compare the activation patterns across participants^51^. To this end, participant-specific temporal BOLD data were first spatially normalized to the fsaverage template. To account for the difference in sampling rate (see MRI data acquisition), we adjusted the fMRI data of three participants which was collected with TR = 0.7 s. We downsampled their data to 1.016 Hz to match the data of the remaining participants, which was collected with TR = 1.016 s. The downsampling was performed using the Python library resampy (see https://github.com/bmcfee/resampy). For each participant, the temporal correlation between the participant’s time course and the average of all other participants’ time courses was computed at each node of the fsaverage surface. This procedure provided a distribution of correlations across participants at each node of the fsaverage surface. The node-wise correlation distributions were then averaged across participants at each node to generate a group ISC map. Prior to averaging, the correlation values were Fisher z-transformed, averaged, and inverse Fisher-transformed^51^. This ISC map allowed us to examine the activation similarity between participants across the cortex.

### Movie annotations

The movie stimulus was manually annotated to detect and label face areas, emotions, and head pose. Face areas were defined as the regions of the video frame that contained a face. For each detected face, annotations included the corresponding pixels in a frame, the identity of the movie character depicted there, and the orientation of the face. Emotions expressed by each character seen in a frame were labeled using six emotion categories: afraid, angry, happy, neutral, sad, and surprised. The head pose was labeled as one of nine categories: left-45, left-90, right-45, right-90, back, front, looking-down, looking-up, and occluded. These annotations of face attributes were provided for every video frame in which a face was detected. Annotations were performed by two independent annotators and discrepancies between the annotators were resolved through discussion and consensus.

Scene cuts in the video were also manually annotated. Scene cuts were defined as a quick pixel-wise transition between two consecutive shots accompanied by a change in video content or camera angle. Annotations included the start and end time of each scene cut, as well as the type of cut (e.g., cut, dissolve, fade-out/in). Scene cuts were also categorized into two types based on manual annotations: continuity cuts and scene changes. Continuity cuts are changes in the camera angle or view, where there is no significant change in the movie content or location; the subsequent scene flows smoothly from the previous one, maintaining narrative continuity. Scene changes are transitions that involve a noticeable shift in content or setting, with the new scene introducing different characters, locations, or events, leading to a distinct break in the narrative or visual presentation. The movie contained 80 continuity cuts and 13 scene changes.

### LFP and iEEG data processing

The data from microwires and macroelectrodes underwent several processing steps before validating data quality. First, a notch filter (zero-double phase, FIR filter at 60 Hz and its harmonics) was applied to attenuate power-line noise. Next, a high-pass filter with a cut-off frequency of 0.1 Hz was used to remove slow fluctuations and drifts in the signal. The channel data were then re-referenced to the common average signal to eliminate common noise and trends. Following re-referencing, time-frequency wavelet decomposition was performed using the Morlet transform with five wavelet cycles for frequencies within the 70 to 170 Hz range, spaced by 10 Hz increments. The power of the signal in each 10 Hz frequency band was z-scored across time to partially correct for the 1/frequency decay of signals. Finally, z-scored power estimates were averaged across frequency bands to obtain a single high-frequency broadband (HFB) time-course per channel.

## Data Records

### Electrophysiology

All data collected in the EMU (electrophysiology, eye tracking, and behavioral recognition ratings) were standardized following the NWB data format^18^. We followed the description of the fields we used from NWB for our data that we published previously^52^. Each NWB file includes various types of data: (1) spike times of all sorted neurons; (2) the LFP from all microwires, which were downsampled to 1000 Hz using decimation, after applying an anti-aliasing low-pass filter^53^ set at 500 Hz. Notably, the data was acquired with a 0.1 Hz high-pass filter, resulting in our LFP/iEEG data being bandpass filtered at 0.1–500 Hz; (3) the field potentials from all iEEG macroelectrodes, similarly downsampled to 1000 Hz; (4) behavior; (5) electrode locations; (6) spike sorting quality metrics; and (7) eye tracking data. The full dataset^54^ is available on DANDI as Dandiset 000623.

### Synchronization (electrophysiology)

TTL pulses were sent from the stimulus presentation computer to the intracranial recording system and the eye tracker to synchronize the three different clocks in these three systems to events. In each system, TTLs were written in a log file together with timestamps of the individual system’s own clock and additional task-specific information such as the number of the specific frame displayed during movie watching or the value of a key pressed during the recognition memory task. The following TTL values were used: start of experiment block = 61, end of experiment block = 66, start of the movie = 4, end of the movie = 10, instruction screen for the recognition memory task = 52, key press to pass the instruction screen or to record the recognition task confidence rating = 33, start probe for a recognition memory trial (image presentation onset) = 7, start of inter-trial interval (ITI) between the key press and the next recognition trial = 9. In addition, during movie watching, a TTL signal was sent every second (cycling through the numbers from 40 to 49 every ten seconds) to serve as a time marker to log the frame number at each second throughout the movie duration. Furthermore, the eye tracker recorded the timestamp corresponding to each frame displayed during the movie watching phase. These timestamp logs allowed precise alignment of brain activity with specific moments in the movie or the recognition memory task, enabling accurate analysis of neural responses to the stimuli and tasks. At the beginning of the movie and after every second of movie watching, TTLs were sent immediately after the first frame of that part of the movie was displayed (as indicated by psychophysics toolbox). Therefore, the maximal uncertainty with respect to the TTL of what is shown on the screen is ~16 ms.

### Structural and Functional MRI

The raw DICOM MRI data, including high resolution structural, field mapping, and BOLD functional data, was converted and organized according to the BIDS data format^19^. Deidentified MRI data^55^ (with coded subject IDs, scan date removed, month and year of birth only in metadata, voxelated faces in structural images) are available on OpenNeuro as dataset ds004798.

### Synchronization (fMRI)

The movie presentation was synchronized to the MRI acquisition using a TTL trigger generated by the pulse sequence for the first slice of the first acquired volume. This trigger signal was converted into a synthetic keypress, which was captured by the stimulation control software (Psychophysics Toolbox). Once the movie was triggered, its frame display was accurately controlled by the stimulation script. The script generated a log file that recorded the frame count every second during the video presentation, similar to how electrophysiology synchronization was handled above.

### Annotations

As described above, we provide the following annotations for the movie: (i) face annotations, including face areas, emotions, and head pose; (ii) scene cut annotations, including time information and the type of the cut (see Movie annotations above). The annotations regarding various face attributions and scene cuts are organized in a pickle format and a CSV format file, respectively. The annotation files^20^ are available on Figshare.

## Technical Validation

### Number of neurons isolated and spike-sorting quality metrics

We recorded 1450 neurons in total from the amygdala, hippocampus, vmPFC, ACC, and preSMA across 29 sessions involving 16 subjects (Fig. 2h). There were on average 1.99 units per wire (only counting wires with at least one isolated neuron) and the mean firing rate of neurons was 2.47 ± 3.32 (mean ± standard deviation, s.d.; Fig. 2a,b). We assessed spike sorting quality using our standard set of metrics^56–58^ (see Fig. 2a–g): (i) the percentage of interspike intervals (ISIs) below 3 ms was 0.40% ± 0.53% (Fig. 2c), showing that neurons were well isolated without refractory period violations; (ii) the ratio between peak amplitude of the mean waveform of each cluster and the standard deviation of the noise was 5.95 ± 3.48 (Fig. 2d; peak SNR) and the mean SNR was 2.07 ± 1.13 (Fig. 2e); (iii) the pairwise projection distance in clustering space between all neurons isolated on the same wire was 11.98 ± 8.93 (Fig. 2f, projection test; in units of s.d. of the signal); and (iv) the median isolation distance was 24.90 (Fig. 2g). The location of each neuron is indicated in MNI152 coordinates and illustrated in Fig. 2i.

### Eye tracking data quality (behavior)

**Fig. 3:**
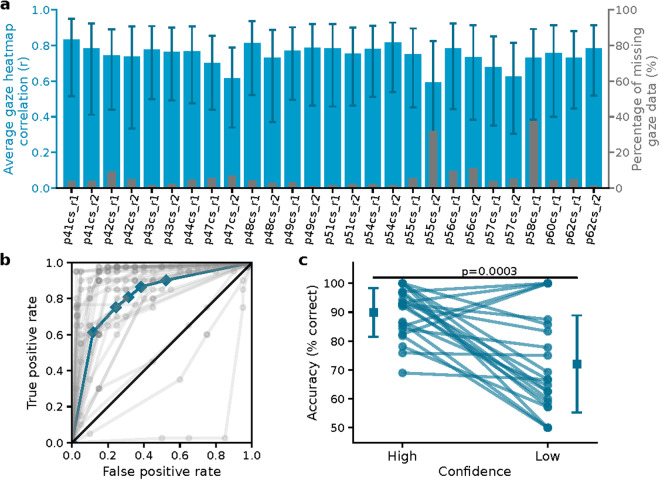
Eye tracking data quality during movie watching and behavioral ROC curves for recognition task for the EMU sessions. (**a**) Eye tracking data quality was assessed using two measures: the correlation between each participant’s gaze heatmap and an average gaze heatmap generated from all participants’ gaze data (blue bar chart), and the percentage of missing gaze data (gray bar chart). Higher correlation values and lower percentages of missing data indicate better quality. Error bars show the standard deviation of correlations across time bins, calculated from the 16^th^ and 84^th^ percentiles of z-transformed correlation values. (**b**) Behavioral ROC curves for each individual session (grey) and the average across all sessions (blue) for the recognition task. Each dot represents a different confidence level, with the highest confidence level (6) corresponding to the point with the lowest false alarm rate. (**c**) Relationship between confidence and accuracy for recognition task. Each line shows the accuracy of responses for high- and low-confidence trials for each individual session. The vertical lines show the standard deviation across all sessions. Accuracy was significantly different between high- versus low-confidence trials (p < 0.001, paired t-test).

The quality of eye tracking data was assessed in two ways. First, the percentage of missing gaze data (either missing data points from the eye tracker or gaze out of the stimulus presentation monitor) was computed for each participant. The gaze data was missing at more than half of the movie watching duration only for one participant (P53CS, the percentage of missing data was 74% in the first run and 84% in the second run of the movie watching phase). The gaze data from this participant was excluded from the eye tracking data quality analysis. Second, temporally binned heatmaps were used to measure the similarity of each participant’s gaze pattern to a reference heatmap generated from the aggregate gaze data of all participants, and both were constructed using gaze data from 1-s epochs of the movie. The reference heatmap represented the visual salience of the movie at the respective time bin. The Pearson correlation was used to compute the similarity between the heatmaps at each time bin and the correlation values were averaged across all time bins for each participant. We found that the individual participant’s gaze patterns were highly correlated with the reference visual salience (Pearson’s r = 0.75 ± 0.12, mean ± s.d., across participants and runs, correlations were Fisher z-transformed prior to averaging, and then the average and s.d. were inverse Fisher-transformed) while having low percentages of missing gaze data (7% ± 8%) (Fig. 3a). These eye tracking results are consistent with previously reported data in healthy subjects^29^ and suggest that the participants were highly engaged with the movie stimulus during the experiment.

### New-old task performance (behavior) in EMU

To obtain event-based neurophysiological data that complement the movie data (cf. Introduction) and also to help assess whether participants paid attention to the movie, we asked participants to perform a recognition memory test after they watched the movie. Participants provided new/old ratings on a confidence scale from 1 (new/novel, not seen during movie watching, sure) to 6 (old/familiar, seen during movie watching, sure; Fig. 1b). Participants had excellent memory: their average accuracy across all confidence levels was 0.79 ± 0.19 AUC (Fig. 3b). In addition, there was no significant difference in memory accuracy between the first and second run for the 13 participants who performed the two runs of movie watching and recognition memory tasks (run #1: 0.79 ± 0.14; run #2: 0.79 ± 0.24 AUC; p = 0.960, paired t-test). Furthermore, participants provided accurate confidence judgments, with accuracy significantly higher for high compared to low confidence judgments (Fig. 3c). Together, this behavioral data indicates that participants attentively watched the movies and that they formed declarative memories^59^ for the content of the movies.

### Imaging data quality

**Fig. 4:**
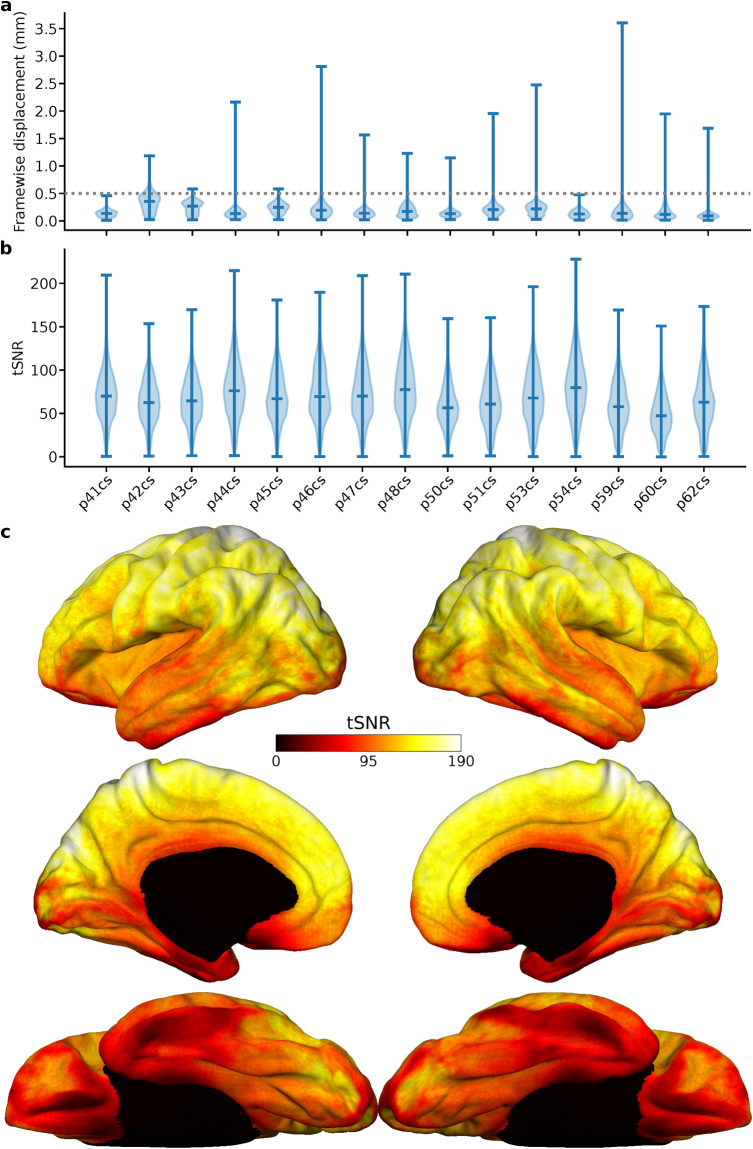
Assessment of fMRI data quality. (**a**) Framewise displacement in mm for each participant across all runs. The violin plots show the minimum, median, and maximum values, along with the distribution of underlying values. (**b**) Whole-brain tSNR distributions for each participant, computed using a brain mask that was generated from each participant’s native anatomical space. The tSNR values were computed for individual voxels in this mask and then averaged across the two runs. (**c**) Mean whole-brain tSNR across runs and participants, computed using data that was projected onto the *fsaverage* template surface.

To validate the fMRI data, we examined various metrics that assess data quality for motion artifacts, signal-to-noise ratio, and consistency across individual participants. Firstly, we examined participant motion during the scans, quantifying framewise displacement and identifying motion outliers. Based on the motion parameters estimated by fMRIprep^30^, we found that participants exhibited minimal motion, with a low median framewise displacement of 0.15 mm (minimum median across participants was 0.09 mm, maximum was 0.38 mm, see Fig. 4a), and a small percentage of volumes (median = 1.8%; min 0.2%, max 14.8%) marked as motion outliers by fMRIprep.

Next, we investigated the temporal signal-to-noise ratio (tSNR) as a measure of data quality across the whole brain and across different cortical areas. We estimated tSNR for each participant in both the participant’s anatomical space (to minimize potential interpolation effects) and the fsaverage template space. The mean whole-brain tSNR across participants was 67.56 ± 8.29, mean ± s.d., consistent with previous datasets^49,60^, suggesting robust signal quality (see Fig. 4b). In addition, we observed expected variations in tSNR across brain regions, with higher values in dorsal areas and lower values in anterior temporal and orbito-frontal cortex and subcortical regions arising from the receive coil sensitivity profile and local signal dropout due to static field inhomogeneities (see Fig. 4c).

**Fig. 5:**
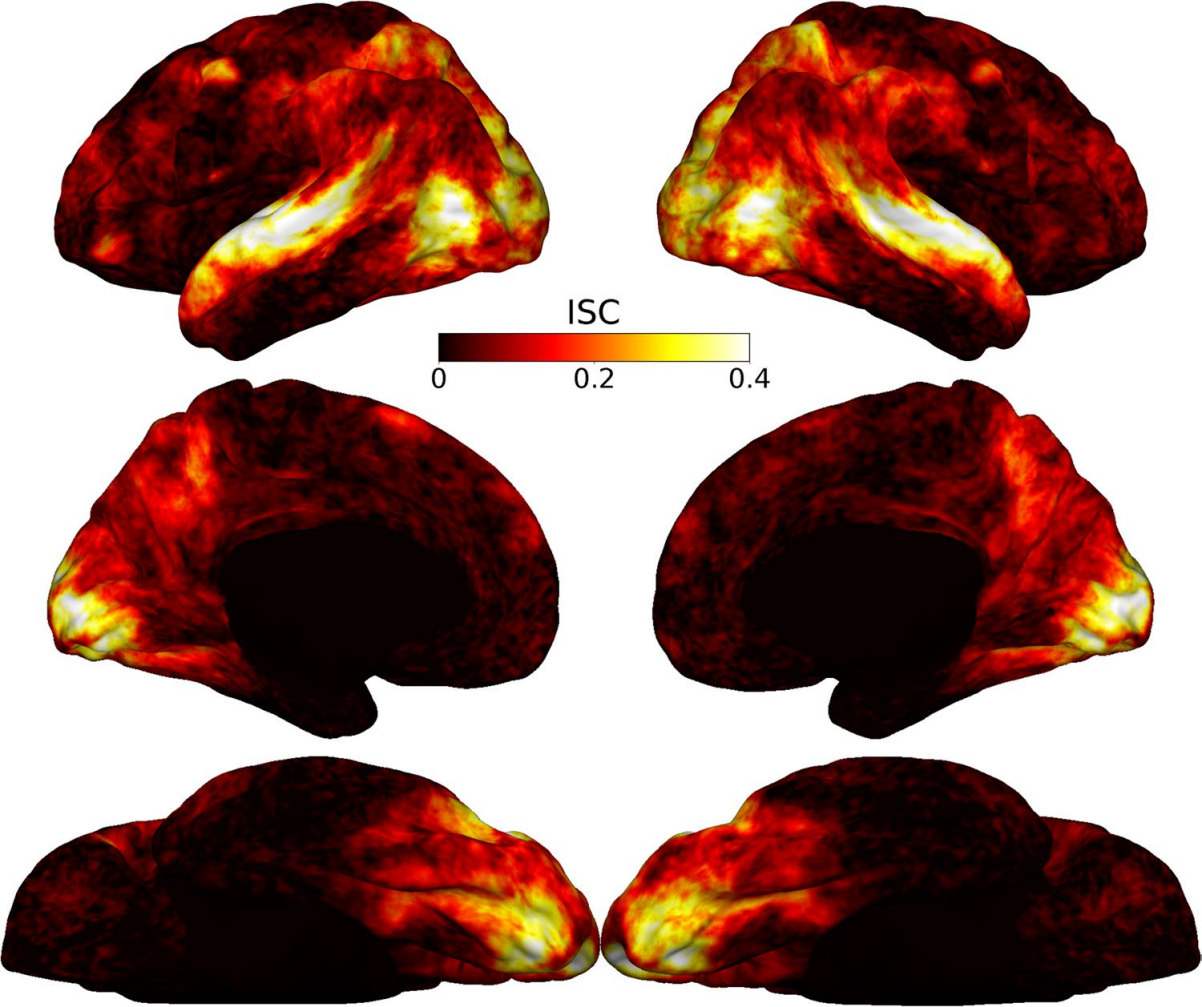
Functional inter-subject correlation. Functional Inter-subject Correlation (ISC) maps of BOLD responses during movie watching. The analysis reveals consistent activation in visual and auditory areas, as well as in regions associated with social cognition (such as precuneus, medial prefrontal cortex, and temporo-parietal junction) and higher-order cognitive processes (such as prefrontal areas), highlighting the responses to complex, real-world stimuli, particularly those with social relevance.

Finally, we used the Inter-subject Correlation (ISC) metric^51^ to assess the consistency of brain responses to the movie stimulus across participants (see Fig. 5). The ISC analysis confirmed that the stimulus elicited comparable brain responses among participants, showing high ISC values in visual and auditory areas as expected for an audio-visual movie stimulus. Furthermore, brain regions associated with processing social information, such as the precuneus, temporo-parietal junction (TPJ), and medial prefrontal cortex (MPFC), demonstrated elevated ISC values. This suggests that the movie content effectively engaged participants in processing social aspects of the narrative.

### Neuronal responses (recognition memory phase)

**Fig. 6:**
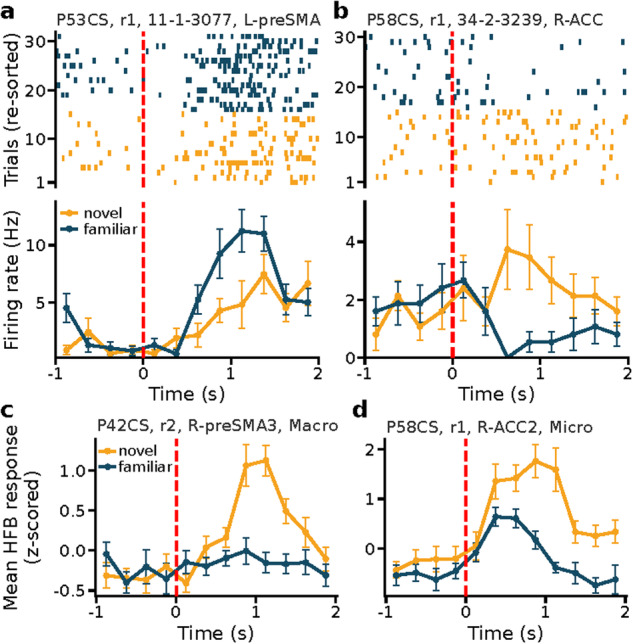
Example memory selective neurons and channels. (**a,b**) Responses during the recognition task from two sample memory neurons located in (**a**) the pre-supplementary motor area (preSMA), showing a firing increase for familiar stimuli (familiarity selective) and (**b**) anterior cingulate cortex (ACC), showing a firing increase for novel stimuli (novelty selective). Top, raster plot; bottom, PSTH (bin size = 250 ms). Trials are aligned to stimulus onset (red line) and ordered by stimulus type (novel versus familiar) for illustration purposes. Error bars indicate the standard error of the mean values. (**c,d**) Average HFB responses from two sample channels recorded from a macroelectrode in the preSMA and a microwire in the ACC, respectively; both showing a response increase for novel stimuli (bin size = 250 ms). Significance of selection criteria (bootstrap test, novel versus familiar) was p = 0.003 (**a**), p < 0.001(**b**), p < 0.001 (**c**), p < 0.001 (**d**).

To validate the neural data, we examined the neural correlates of novelty/familiarity during the recognition memory task. We only used correct trials for this analysis, i.e., we compared true positives (familiar) with true negatives (novel). Note that this analysis is restricted to recognition and does not present any analysis of signals at encoding. We referred to cells or channels as ‘memory selective’ if their response (average firing rate or HFB power, respectively) in a 1.5 s window starting 200 ms after stimulus onset differed significantly between novel and familiar trials (bootstrap comparison of means with 10,000 runs, p < 0.05). Of the overall 1450 recorded neurons, 1266 were included in this analysis. The remainder was excluded based on these behavioral criteria: i) behavioral AUC > 0.6 on all trials, ii) less than 10 correct trials per condition^59^. Excluded patients and runs were P44CS run 1, P51CS run 2, P56CS run 2, P57CS runs 1 and 2. Significant proportions of cells were memory selective in the amygdala (32/436, p = 0.015, permutation test with 2,000 shuffling of average firing rates across trials for each cell), ACC (22/186, p = 0.002), and vmPFC (14/150, p = 0.016) but not the hippocampus (14/238, p = 0.30) and preSMA (17/256, p = 0.17). The properties of the selected memory selective cells are comparable to those in similar tasks in published work (Fig. 6a,b show examples), including the finding that memory selective cells are more common in the amygdala compared to the hippocampus in some tasks^1,26^. We next repeated the same analysis using HFB power for the local field potentials recorded on the microwires as well as the macroelectrodes that were in the same target structures. For the LFP, significant proportions of channels were memory selective in the amygdala (37/368, p = 0.002, permutation test with 2,000 shuffling of average HBF power across trials for each channel), ACC (33/368, p = 0.028), and preSMA (40/368, p = 0.001) but not hippocampus (17/368, p = 0.90) and vmPFC (28/336, p = 0.05). At the iEEG level (macro channels), significant proportion of channels in the ACC (41/368, p < 0.001), vmPFC (34/368, p = 0.012), and preSMA (45/368, p < 0.001) but not the amygdala (25/368, p = 0.47) and hippocampus (32/368, p = 0.067) were memory selective for HFB power (Fig. 6c,d show examples). Together, this analysis confirms that memory-selective signals were present at both the single-neuron and LFP level. We note that in this analysis we only used correct trials and we are therefore not differentiating between decision and memory signals^26^.

The brain areas for which significant percentages of memory-selective cells and/or LFP/iEEG channels were found varied by signal modality. For example, the ACC showed memory selectivity in all three modalities, the hippocampus showed selectivity in none of them, and the amygdala showed selectivity at the single-cell and LFP but not iEEG level. These discrepancies indicate that these three signal modalities are distinct, an aspect that our dataset is well suited to investigate. Lastly, we hypothesize that the absence of significant memory selectivity in the hippocampus in all three signal modalities is due to a lack of statistical power because we have seen before that memory selective cells in the hippocampus are weaker and less common than those in the amygdala in some tasks^26^.

### Neuronal responses (movie watching phase)

**Fig. 7:**
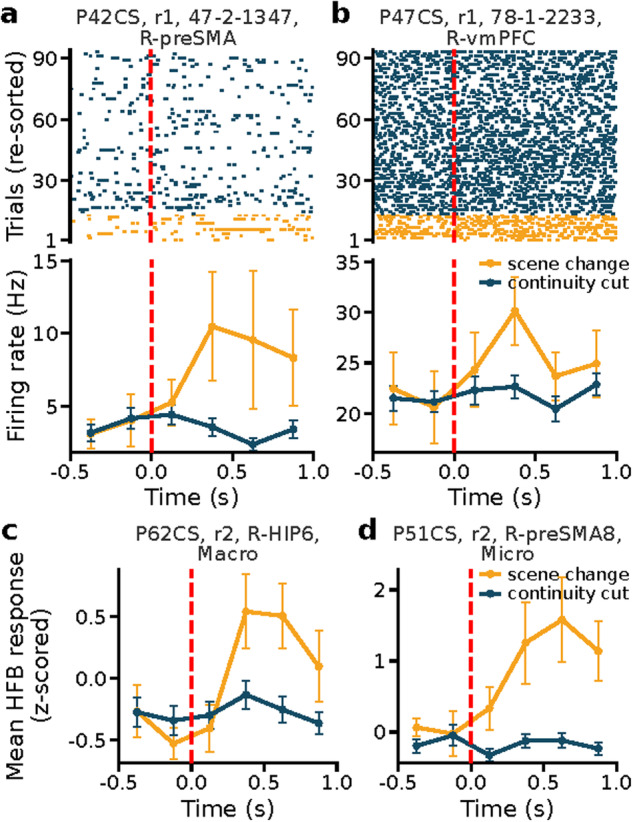
Example event selective neurons and channels. (**a,b**) Responses during the movie watching from two sample event neurons located in (**a**) the pre-supplementary motor area (preSMA) and (**b**) ventral medial prefrontal cortex (vmPFC). Top, raster plot; bottom, PSTH (bin size = 250 ms). Trials are aligned to scene cut start (red line) and ordered by scene cut type (scene change versus continuity cut) for illustration purposes. Error bars indicate the standard error of the mean values. (**c,d**) Average HFB responses from two sample channels that showed differential responses to scene changes compared to continuity cuts. The responses were recorded from a macroelectrode in the hippocampus and from a microwire in the preSMA, respectively (bin size = 250 ms). Significance of selection criteria (bootstrap test, scene change versus continuity cut) was p = 0.024 (**a**), p = 0.030 (**b**), p = 0.026 (**c**), p < 0.001 (**d**).

To validate the neural data recorded during the movie watching phase, we analyzed the neural responses to two types of scene cuts: continuity cuts and scene changes (see Movie Annotations). Continuity cuts refer to changes in the camera view without significant alterations in the movie content or location, while scene changes involve noticeable shifts in content or setting, leading to new episodes (events) in the movie. We referred to cells or channels as ‘event selective’ if their response (average firing rate or HFB power, respectively) in a 1.0 s window starting from a scene cut onset differed significantly between scene changes and continuity cuts (bootstrap comparison of means with 10,000 runs, p < 0.05). Significant proportions of cells were event selective in the vmPFC (10/175, p = 0.005, permutation test) and preSMA (20/299, p < 0.001) but not in the amygdala (15/479, p = 0.081), ACC (7/236, p = 0.224), and hippocampus (8/261, p = 0.199; Fig. 7a,b show examples). We next repeated the same analysis using HFB power for the local field potentials recorded on the microwires as well as the field potentials from the macroelectrodes that were in the same target structures. For the LFP, significant proportions of channels were event selective in the amygdala (20/448, p = 0.005, permutation test), hippocampus (40/448, p < 0.001), ACC (33/448, p < 0.001), vmPFC (15/400, p = 0.015), and preSMA (22/448, p = 0.001). At the iEEG level (macro channels), significant proportion of channels in the amygdala (67/448, p < 0.001), hippocampus (41/448, p < 0.001), ACC (25/448, p < 0.001), vmPFC (33/449, p < 0.001), and preSMA (21/448, p = 0.002) were event selective (Fig. 7c,d show examples). Together, this analysis confirms that event selective signals were present at both the single-neuron and LFP level.

## Usage Notes

The NWB files are labelled by patient ID and session number. For manipulating an NWB file in Python, such as reading the file in and using the fields and functions associated with NWB classes, see the provided code in the GitHub repository (see Code Availability) and the pynwb module documentation^18^. All single-unit spike times (found in NWBFile.units) are referenced to the movie start. Note that LFP samples (found in NWBFile.processing[‘ecephys’]) from 10 s before movie start are included for baseline and to reduce edge effects in time-frequency analyses. To create timepoints for the LFP samples, start at 10 seconds before the starting time (ElectricalSeries.starting_time) and increment by 1/sampling frequency (ElectricalSeries.rate, 1000 Hz). Samples taken during movie watching start at 10 s. Spike detection was conducted in blocks of 16 seconds^25^, with the spike detection threshold determined based on the standard deviation of the signal in that block. If there was high-amplitude noise or an artifact in a given block, it is possible that no spikes are detected in an entire 16-second-long block. This effect can be seen in raster plots for certain units and does not mean the unit did not fire during that time block. For the exact time points of frames in a given session, such as finding the time of a frame when a scene cut occurs, we recommend using the times found in NWBFile.stimulus[“movieframe_time”]. The movie has a frame rate of 25 Hz. Depending on the Python package used to load the movie, the number of frames may differ. We recommend using a package such as OpenCV for a total of 11971 frames. For the time points of experiment events, such as when the movie ended, we recommend using the times found in NWBFile.trials. Note that there was a variable delay between the end of the movie and the start of the recognition trials, but electrophysiology data is still included for that time in between these two events. To determine in which brain area a particular unit is located, we recommend taking the electrode ID from NWBFile.units and looking up the electrode location in NWBFile.electrodes. It is helpful to convert both the NWBFile.units and NWBFile.electrodes into pandas DataFrames. For anonymity, the session dates (NWBFile.session_start_time) are not the actual dates of the sessions. All structural MR images are deidentified by irreversible face voxelation using voxface (https://github.com/jmtyszka/voxface).

## Data Availability

All code is available in the GitHub repository (https://github.com/rutishauserlab/bmovie-release-NWB-BIDS). The python code includes scripts to read and plot the data from the NWB files and perform the analyses presented in this data descriptor. The code relies heavily on open-source Python packages such as numpy^61^, scipy^62^, pynwb^18^, mne-python^63^, nilearn^48^, and pycortex^64^. The movie annotation files are also provided in the GitHub repository under ‘assets/annotations’ folder. The scripts related to the estimation of tSNR and ISC were adapted from the code provided in the GitHub repository associated with the budapest-fmri-data study^49,50^ (see: https://github.com/mvdoc/budapest-fmri-data).
